# The recombination landscapes of spiny lizards (genus *Sceloporus*)

**DOI:** 10.1093/g3journal/jkab402

**Published:** 2021-11-22

**Authors:** Cyril J Versoza, Julio A Rivera, Erica Bree Rosenblum, Cuauhcihuatl Vital-García, Diana K Hews, Susanne P Pfeifer

**Affiliations:** 1 School of Life Sciences, Arizona State University, Tempe, AZ 85281, USA; 2 Center for Evolution and Medicine, Arizona State University, Tempe, AZ 85281, USA; 3 Department of Environmental Science, Policy and Management, University of California, Berkeley, Berkeley, CA 94720, USA; 4 Departamento de Ciencias Veterinarias, Programa de Maestría en Ciencia Animal, Universidad Autónoma de Ciudad Juárez México, Chihuahua 32315, Mexico; 5 Department of Biology, Indiana State University, Terre Haute, IN 47809, USA; 6 Center for Mechanisms of Evolution, Arizona State University, Tempe, AZ 85281, USA

**Keywords:** recombination, spiny lizards, *Sceloporus jarrovii*, *Sceloporus megalepidurus*

## Abstract

Despite playing a critical role in evolutionary processes and outcomes, relatively little is known about rates of recombination in the vast majority of species, including squamate reptiles—the second largest order of extant vertebrates, many species of which serve as important model organisms in evolutionary and ecological studies. This paucity of data has resulted in limited resolution on questions related to the causes and consequences of rate variation between species and populations, the determinants of within-genome rate variation, as well as the general tempo of recombination rate evolution on this branch of the tree of life. In order to address these questions, it is thus necessary to begin broadening our phylogenetic sampling. We here provide the first fine-scale recombination maps for two species of spiny lizards, *Sceloporus jarrovii* and *Sceloporus megalepidurus*, which diverged at least 12 Mya. As might be expected from similarities in karyotype, population-scaled recombination landscapes are largely conserved on the broad-scale. At the same time, considerable variation exists at the fine-scale, highlighting the importance of incorporating species-specific recombination maps in future population genomic studies.

## Introduction

In most sexually reproducing organisms, recombination is critically important. On the one hand, recombination ensures the proper pairing and segregation of homologous chromosomes during meiotic cell division; on the other, it creates novel combinations of alleles through the exchange of genetic material between the parental chromosomes upon which selection may act (Hill and Robertson 1966; Felsenstein 1974; Felsenstein and Yokoyama 1976; Otto and Barton 2001). Recombination also plays a pivotal role in shaping the spatial distribution of variation within a genome and modulating genetic diversity among individuals (Maynard Smith and Haigh 1974; Begun and Aquadro 1992; Charlesworth *et al.* 1993), facilitating adaptation to novel or changing environments (Charlesworth 1976), and contributing to the formation of new species (Nachman and Payseur 2012; Payseur 2016). Moreover, as recombination rate variation influences the performance of genome scans to identify signatures of positive selection (Booker *et al.* 2020), a detailed knowledge of recombination landscapes is essential for many ecological and evolutionary studies.

Recombination is a quantitative, heritable trait subject to selection (Charlesworth and Charlesworth 1985) that may exhibit plasticity due to environmental or physiological factors (Stevison *et al.* 2017). Although constraints appear to exist with regards to an organismal minimum and maximum recombination rate—imposed by an obligate crossover per chromosome (or chromosome arm) and by crossover interference, respectively (see review by Ritz *et al.* 2017 and references therein), tremendous variation in rates and patterns of recombination exists across the tree of life—between species, populations, and individuals—as well as across the genome (see review by Stapley *et al.* 2017 and references therein). Yet, relatively little remains known about rates of recombination in the vast majority of species, including squamate reptiles—the second largest order of extant vertebrates.

*Sceloporus* is a diverse genus of lizards native to North America with roughly 100 species that have been well-studied in terms of behavior (Hews and Martins 2013), habitat (Lawing *et al.* 2016; Rivera *et al.* 2020, 2021), and phylogenetic relationships (Wiens *et al.* 2010; Lambert and Wiens 2013; Leaché *et al.* 2016). *Sceloporus* species display an unusual variability in chromosome number—ranging from 22 to 46 chromosomes (Sites *et al.* 1992), resulting in a rapid differentiation among species with markedly different chromosome counts (Leaché *et al.* 2016). Differences in chromosome number may not only promote speciation (Hall 2009), they also impose significant constraints on genome evolution (Bedoya and Leaché 2021) and may lead to changes in broad-scale recombination landscapes (Dumas and Britton-Davidian 2002). Here, we use population genetic data to characterize and compare genome-wide recombination profiles of *Sceloporus jarrovii* and *Sceloporusmegalepidurus—*two vivaparous species that diverged at least 12 Mya and for which similar constraints on recombination might be expected due to their belonging to the same 32-chromosome clade (Leaché *et al.* 2016).

## Materials and methods

### Population sampling

We captured eight *S.jarrovii* and eight *S. megalepidurus* individuals (four males and four females per species) in the field during peak breeding season in early October 2013 in south-eastern Arizona, United States, and in late August 2013 near Veracruz, Mexico, respectively (Supplementary Figure S1). Lizards were initially placed in uniquely numbered cloth bags and later sacrificed by first cooling individuals over ice and then rapidly decapitating (IACUC protocols 492636-1 and 962836-1 to DKH). Liver samples were collected from each individual and placed in RNAeasy solution. Samples were held in a −5**°**C freezer while in the field (for 2–3 weeks) until permanently stored in a −20**°**C freezer at Indiana State University.

### DNA extraction, library preparation, and sequencing

DNA was extracted from the liver samples at the Yale Center for Genomic Analysis following the chemagic^™^ DNA Tissue100 H24 prefilling VD1208504.che protocol (PerkinElmer Ref# CMG-1207). Specifically, tissue samples were lysed overnight in 1 ml chemagic^™^ lysis buffer and 50 µl Proteinase K at 56**°**C. The next day, samples were treated with 80 µl RNase A at 4 mg/µl (AmericanBio Ref# AB12023-00100) for 10 min at 56**°**C before transferring the lysates into deep well plates. DNA was extracted using the chemagic^™^ 360 Nucleic Acid Extractor (PerkinElmer). Next, samples were transferred to intermediate tubes and centrifuged at 13,000 rpm for one minute, placed on a magnet, and transferred to final tubes.

To ensure that the quantity and quality of the extracted DNA were sufficient for sequencing, DNA concentration was measured using a Qubit fluorometer (Thermo Fisher) and purity assessed by measuring 260/280 nm and 260/230 absorbance ratios on a NanoDrop. DNA was fragmented using a Covaris E220 Focused-ultrasonicator, and size-selected to an average length of 350 bp. Fragmented DNA with 3′ and 5′ overhangs was purified and dual-size selected using Agencourt AMPure XP magnetic beads. T4 DNA Polymerase and Polynucleotide Kinase were used to repair the ends of the DNA fragments to which Illumina TruSeq UD Index adapters were subsequently ligated to allow for hybridization to the flow cells.

Libraries were sequenced on an Illumina NovaSeq 6000 platform following manufacturer's protocols. Signal intensities were converted to base calls using the platform's proprietary real time analysis (RTA) software. To monitor quality during sequencing, Illumina's Phi X library was spiked into each lane at a concentration of 1% as a positive control. Last, samples were de-multiplexed using CASAVA v.1.8.2.

### Reference assembly

Generating *de novo* assemblies remains a time-consuming and expensive endeavor. At the same time, mapping reads from individuals of one species to the genome of another, distantly related species can pose several challenges (see discussion in Pfeifer 2017). To avoid biasing our analyses toward one of the two focal species and allow for fair genomic comparisons, a genome assembly was generated from a third species, *Sceloporus cowlesi*, which is equally closely related to both *S. jarrovii* and *S. megalepidurus* (Leaché *et al.* 2016). For this purpose, tissue from a single *S. cowlesi* individual collected at White Sands National Park (Otero County, NM) was used for high molecular weight (HMW) DNA extraction, 10X Genomics Chromium Genome library preparation, and sequencing on an Illumina HiSeq 4000. To create a draft genome assembly, raw sequence reads were processed for quality assurance using a custom in-house pipeline, proc10xG (https://github.com/ucdavis-bioinformatics/proc10xG; last accessed November/3rd 2021), together with the Kmer Analysis Toolkit (Mapleson *et al.* 2017). Specifically, 10X gem barcodes were checked for expected distribution and a genome *k*-mer analysis was performed to estimate genome size, repeat content, and other genomic features. Next, Supernova v.2.1.1 (Weisenfeld *et al.* 2017) was used to generate a *de novo* assembly, using ∼826 million single raw reads as input (default settings). As the assembly algorithm is designed to work specifically with data generated using the 10X Genomics Chromium system, no additional processing of sequencing reads was necessary. The resulting reference assembly contained 34,570 scaffolds with an overall length of 1.91 Gb (N50 = 62,759,035 bp as determined by QUAST v.5.0.2; Mikheenko *et al.* 2018).

### Sequence alignment

To check initial data quality, raw sequence reads were visualized using FastQC v.0.11.7 (http://www.bioinformatics.babraham.ac.uk/projects/fastqc; last accessed 27 March 2021) and adapters and low-quality regions subsequently trimmed using Trim Galore! v.0.6.1 (http://www.bioinformatics.babraham.ac.uk/projects/trim_galore/; last accessed March/27th 2021). The pre-processed reads were aligned to the *S. cowlesi* reference assembly using BWA mem v.0.7.17 (Li and Durbin 2009) with default parameters. Despite the use of a reference assembly from a different *Sceloporus* species, 96.5% and 96.9% of reads could be mapped for *S. jarrovii* and *S. megalepidurus*, respectively. Aligned reads were validated, sorted, and indexed using SAMtools v.1.9 (Li *et al.* 2009) and duplicates marked using Picard v.2.18.3 (http://broadinstitute.github.io/picard/; last accessed March/27th 2021). Following mapping, the mean per-individual coverage for *S. jarrovii* (*n* = 8) and *S. megalepidurus* (*n* = 8) was 10.4X and 11.2X, respectively (Supplementary Table S1).

### Variant calling, genotyping, and filtering

Variants were called using the Genome Analysis Toolkit (GATK) HaplotypeCaller v.3.7.0 (Poplin *et al.* 2017) and jointly genotyped using GenotypeGVCFs v.4.1.2.0. In the absence of a curated dataset required to train GATK's variant filtration machine learning algorithm, variants were hard-filtered following GATK's Best Practices (Van der Auwera *et al.* 2013; Van der Auwera and O'Connor 2020). Specifically, GATK’s SelectVariants and VariantFiltration v.4.1.2.0 were used to filter out variants with (1) poor alignment characteristics as indicated by low alignment qualities (*i.e.*, QD < 2.0; MQ < 20.0; MQRankSum < –12.5), (2) evidence of strand bias as estimated by Fisher’s exact test (FS > 60.0) or the symmetric odds ratio test (SOR > 3.0), or (3) evidence of a positional bias in read position (ReadPosRankSum < –8.0). In addition, as false-positive variants frequently exhibit excessive read coverage and tend to occur more often in regions where a large number of other variants were called, variants with (5) extremely low (<4X) or high (>15X) read coverage (*i.e.*, DP ≥ 32 && DP ≤ 120) or (5) an extensive clustering (three or more variants within a 10 bp window, *i.e.*, cluster size = 3; cluster window size = 10) were removed. Last, the dataset was limited to biallelic single nucleotide polymorphisms (SNPs) genotyped in all samples of a species (*i.e.*, AN = 16).

Due to the inherent difficulties of reliably calling variants in nonmodel organisms, several sanity checks were performed. First, assuming a constant genome-wide mutation rate, the number of variants on each scaffold should roughly correspond to its length. Although SNP densities agreed well across long scaffolds, a preliminary analysis highlighted a large variation in SNP density (ranging from 0 to 0.035) on the smallest scaffolds, likely due to misaligned reads and artifactual variant calls (Supplementary Figure S2). In order to limit the number of false positives in this study, analyses were thus restricted to scaffolds longer than 2 Mb (*i.e*., a total of 88 scaffolds). Importantly, as these 88 (out of the total 34,570) scaffolds comprise 1.63 out of the 1.91 Gb assembled genome, 94.9% and 95.2% of variants were retained for *S. jarrovii* and *S. megalepidurus*, respectively.

Low-complexity and repetitive regions often result in ambiguous read alignments that can lead to erroneous variant calls (see review by Pfeifer 2017). Consequently, five different classes of repeats—LINEs, LTRs, DNA transposons, simple repeats, and low complexity regions—were annotated using RepeatMasker v.4.1.0 (http://www.repeatmasker.org; last accessed March/27th 2021) and SNPs within these regions were removed from the dataset.

As collapsed copy number variants and other misassembled regions can lead to artifactual excessive heterozygosity in the genome, VCFtools v.0.1.16 (Danecek *et al.* 2011) was used to filtered out SNPs with a *P*-value < 0.01 for Hardy-Weinberg equilibrium.

As a final sanity check, both per-sample coverage and number of variants were compared across scaffolds. As shown in Supplementary Figure S3, with the exception of three scaffolds (90, 90602, and 90921), the per-sample coverage was highly consistent. In addition, the number of variants on each scaffold was highly consistent across samples (Supplementary Figure S4).

The final dataset for *S. jarrovii* contained 5,927,176 segregating sites (with a transition-transversion ratio, TsTv, of 2.05) and 217,678 fixed differences to the *S. cowlesi* reference assembly in the accessible part of the genome (959,437,632 bp). The final dataset for *S. megalepidurus* contained 8,742,115 segregating sites (Ts/Tv = 1.96) and 211,825 fixed differences to the *S. cowlesi* reference assembly in the accessible part of the genome (980,116,223 bp).

### Kinship and population structure

Genetic relatedness among individuals was inferred using the software KING (Manichaikul *et al.* 2010) as implemented in plink2 (Chang *et al.* 2015) (Supplementary Figure S5). Genetic differentiation among individuals was explored using a principal component analysis (PCA; Supplementary Figure S6) and individual ancestries were assessed using the software ADMIXTURE (Alexander *et al.* 2009). As SNPs in linkage disequilibrium (LD) can distort signals of population structure (Liu *et al.* 2020), SNPs were pruned for linkage using plink2 (Chang *et al.* 2015). Specifically, the plink2 command “–indep-pairwise 50 5 0.2” was run, for each 50-SNP window, to exclude one of a pair of SNPs if their pairwise association *r*^2^ > 0.2 (sliding window size: 5 SNPs). After filtering, 277,341 and 337,214 SNPs remained in the LD-pruned *S. jarrovii* and *S. megalepidurus* datasets, respectively. Next, the R package SNPRelate v.1.20.1 (Zheng *et al.* 2012) was used to perform a PCA (Supplementary Figure S6). In addition, ADMIXTURE v.1.3.0 (Alexander *et al.* 2009) was run to infer admixture proportions for 1–4 ancestral source populations (*K*). The best model was chosen to minimize the cross-validation error rates (Supplementary Figure S7). Finally, population genetic summary statistics (nucleotide diversity π and Tajima's *D*) were calculated for each species using VCFtools v.0.1.16 (Danecek *et al.* 2011) and pixy v.1.2.5.beta1 (Korunes and Samuk 2021) on the full dataset.

### Phasing

Following Auton *et al.* (2012), genotypes were phased using PHASE v.2.1.1 (Stephens *et al.* 2001b; Stephens and Donnelly 2003) to reconstruct haplotypes. Specifically, each scaffold was partitioned into 400 SNP regions with a 100-SNP overlap between regions, and regional files in the variant calling format (.vcf) were converted into PHASE input using a modified version of vcf2PHASE.pl (vcf-conversion-tools 1.0; Zenodo: http://doi.org/10.5281/zenodo.10288; last accessed March/27th 2021). Haplotypes were reconstructed using the “recombination model” in PHASE with the following options: “200 1 300 ‐MR ‐F.05 ‐l10 ‐x5 –X5”. Overlapping phased regions were joined back together using a modified version of join_phase_blocks.pl (great-ape-recombination 1.0; Zenodo: http://dx.doi.org/10.5281/zenodo.13975; last accessed March/27th 2021) and converted into linkage format using plink v.1.90b3.45 (Purcell *et al.* 2007). Excluding sites that fixed during phasing, the final phased datasets contained 5,924,882 and 8,685,799 variants with a transition-transversion ratio of 2.05 and 1.96 for *S. jarrovii* and *S. megalepidurus*, respectively.

### Recombination rate estimation

Population recombination rates (*ρ* = 4 *N_e_ r*, where *N_e_* is the effective population size and *r* is the recombination rate per site per generation) were inferred using LDhat v.2.2 (McVean *et al.* 2002, 2004; Auton and McVean 2007)—a method that has been widely applied in the field, including in the only other study of recombination landscapes in lizards (Bourgeois *et al.* 2019), and that is suitable for small sample sizes (Auton *et al.* 2012). As the computation of two-locus coalescent likelihoods is computationally expensive, a likelihood lookup table was calculated to speed up analyses. To this end, LDhat “convert” was used to infer Watterson's infinite-sites estimator of the population-scaled mutation rate (Θ). An approximation of Watterson's Θ of 10^−4^ was then used to generate a likelihood lookup table using LDhat “complete” with a 101-point grid resolution. This lookup table was used to estimate recombination rates in the species following Auton *et al.* (2012). Specifically, each phased scaffold was partitioned into 4000 SNP regions with a 200-SNP overlap between regions. Next, LDhat “interval” was run for 60 million iterations with a block penalty of 5 and samples were taken every 40,000 iterations. After using LDhat “stat” to discard the first 20 million iterations as burn-in, recombination rate estimates were joined at the mid-points of the 200-SNP overlapping regions. Using these estimates, correlations with nucleotide diversity (π) and GC-content were calculated on the 1 Mb-, 500 kb-, and 100 kb-scale.

## Results and discussion

The genomes of 16 wild-caught spiny lizards—eight *S.jarrovii* and eight *S. megalepidurus* (four males and four females per species; Supplementary Figure S1)—were sequenced to an average coverage of 10X per individual (Supplementary Table S1). Quality-controlled reads were mapped to a draft *S. cowlesi* reference genome (34,570 scaffolds, N50 = 62,759,035 bp) and SNPs called following the GATK Best Practices (Van der Auwera *et al.* 2013; Van der Auwera and O'Connor 2020). Analyzing the patterns of variation across samples and scaffolds suggested that SNPs residing on scaffolds smaller than 2 Mb were likely false positives due to misaligned reads and spurious variant calls (Supplementary Figure S2), thus they were discarded in subsequent analyses. Stringent filter criteria were employed to produce high-quality datasets containing 5.9 and 8.7 million biallelic SNPs on the remaining genomic scaffolds (*i.e.*, 88 scaffolds comprising 1.63 out of the 1.91 Gb assembled genome) for *S. jarrovii* and S*. megalepidurus*, corresponding to a SNP density of 3.6 and 5.3/kb, respectively (see *Materials and Methods* for details). Per-sample coverages were relatively evenly distributed across these 88 scaffolds, suggesting that there were no significant issues caused by either the sequencing strategy (*e.g.*, biases introduced by PCR enrichment) or genome assembly (*e.g.*, biases due to extreme base composition) (Supplementary Figure S3). The number of identified variants was consistent across regions—a further indicator that there were no systematic sequencing or mapping errors (Supplementary Figure S4). Moreover, transition-transversion ratios of 2.05 and 1.96 for *S. jarrovii* and *S. megalepidurus* agree well with the genome-wide average of ∼2.0 seen in many organisms (*e.g.*, Stephens *et al.* 2001a). Both species exhibit similar nucleotide diversity levels (*S. jarrovii*: 0.17%; *S. megalepidurus*: 0.32%)—close to the levels of diversity previously reported for different populations of *S. cowlesi* (0.25%–0.27%)—and Tajima's *D-*values ranging from −0.51 in *S. jarrovii* to 0.46 in *S. megalepidurus*, indicating a relatively unskewed site frequency spectrum (*S. cowlesi*: −0.17 to 0.25; Laurent *et al.* 2016).

Genome-wide population-scaled recombination rates for *S. jarrovii* and *S. megalepidurus* were inferred from patterns of LD using the LDhat methodology (McVean *et al.* 2002, 2004; Auton and McVean 2007), which relies on polymorphism data from unrelated individuals. Analyzing patterns of genetic relatedness and differentiation among individuals confirmed that all individuals included in this study were genetically unrelated (Supplementary Figure S5). Although no family relationships were detected among the sampled individuals, negative estimates of pairwise kinship coefficients indicated a putative structuring of the populations. To better understand any population structure potentially present in the samples, genetic differentiation among individuals was explored using a principal component analysis (Supplementary Figure S6) and individual ancestries were assessed using the software ADMIXTURE (Alexander *et al.* 2009). As expected from the sampling design, a single ancestral source population (*K* = 1) provided the best fit to the data whereas models with more than one source population (*K* > 1) led to overfitting (Supplementary Figure S7).

**Figure 1:**
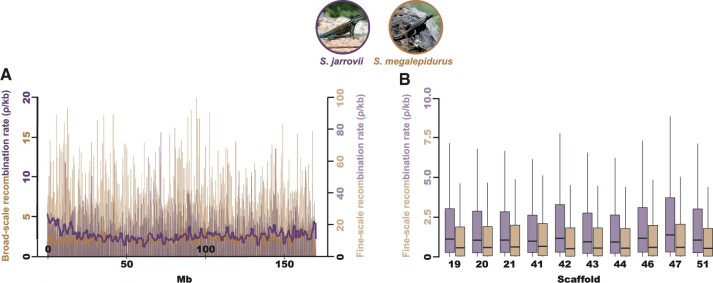
Recombination landscapes in *S. jarrovii* and *S. megalepidurus.* (A) Broad- and fine-scale recombination rates along the longest scaffold (scaffold 19) in *S. jarrovii* (shown in purple) and *S. megalepidurus* (shown in orange). Broad-scale rates were averaged over 1 Mb-regions. (B) Variation in the fine-scale recombination landscape within and between scaffolds in *S. jarrovii* (purple) and *S. megalepidurus* (orange). Only the 10 longest scaffolds are shown here; the 88 scaffolds used in this study are displayed in Supplementary Figures S8 and S9. Picture credits: squamatologist (*S. jarrovii*; distributed under a CC BY-NC-ND 2.0 license) and camamed (*S. megalepidurus*; distributed under a CC BY-NC 4.0 license).

**Figure 2:**
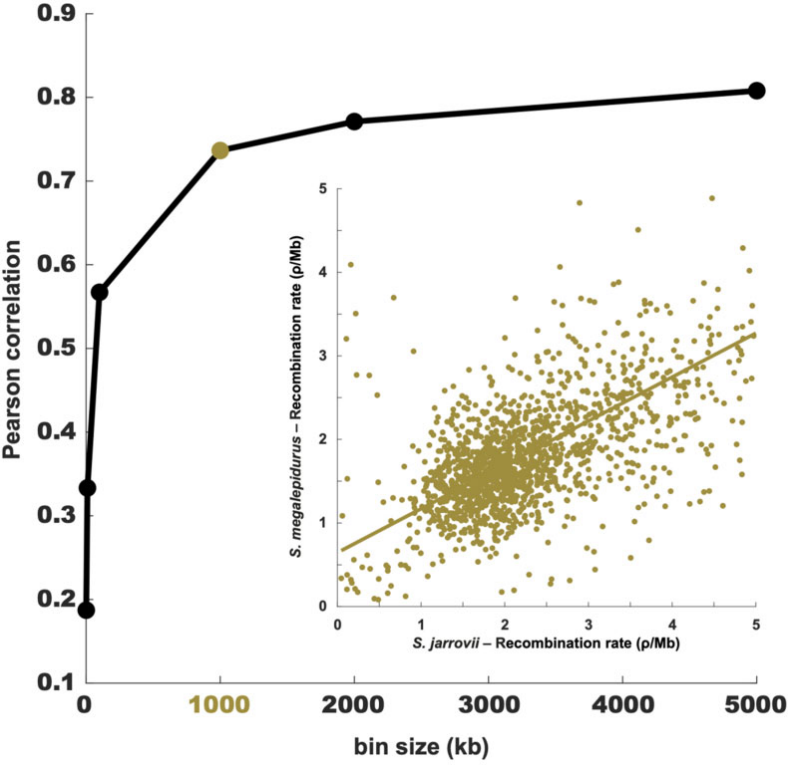
Correlation between the recombination maps of *S. jarrovii* and *S. megalepidurus*. Pearson correlation between the recombination maps of *S. jarrovii* and *S. megalepidurus* at different scales; the inlay shows the correlation at the broad (1 Mb) scale.

**Figure 3:**
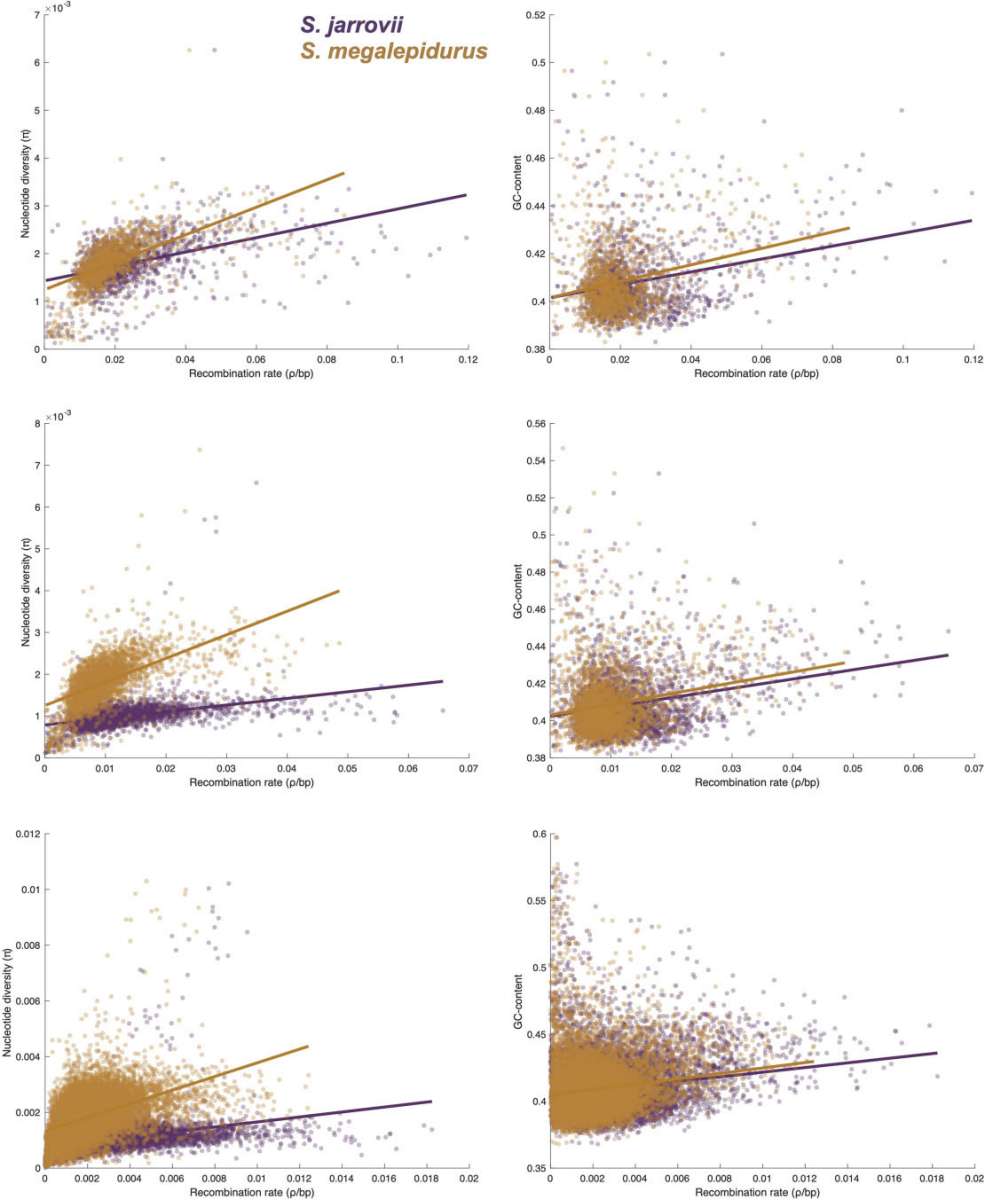
Relationship between genome-wide recombination rate with nucleotide diversity and GC-content. Relationship between genome-wide recombination rate with nucleotide diversity (left) and GC-content (right) in *S. jarrovii* (purple) and *S. megalepidurus* (orange) at the 1 Mb- (top), 500 kb- (middle), and 100 kb- (bottom) scale.

Although population-scaled recombination rates are generally higher in *S. jarrovii* than in *S. megalepidurus* (Figure 1A), recombination landscapes are largely conserved on the broad-scale, with a positive correlation of 0.74, 0.77, and 0.81 on the 1-, 2-, and 5 Mb-scale, respectively (Figure 2). On the fine-scale, considerable variation exists between the two species as well as along their genomes (Figure 1B, Supplementary Figures S8 and S9), with the strength of correlation decreasing with successively smaller scales from 0.57 at 100 kb to 0.19 at 1 kb (Figure 2). However, it is important to note that the variance will be larger at the fine-scale which may (at least in part) drive this observation. Recombination rates in *S. jarrovii* and *S. megalepidurus* are positively correlated with genome-wide nucleotide diversity (Figure 3A) and GC-content (Figure 3B), with observed differences in the shape of the relationship between the two species being likely driven by differences in the underlying effective population sizes. These patterns are in concordance with previous work in many vertebrates—including other squamates (Bourgeois *et al.* 2019; Schield *et al.* 2020)—and are likely caused by the pervasive effects of selection at linked sites (see reviews by Cutter and Payseur 2013; Charlesworth and Jensen 2021) and biased gene conversion (Pessia *et al.* 2012), respectively.

Last, it is important to note the limitations of estimating recombination rates from population-level sequencing data. First, population genetic approaches estimate historical recombination rates averaged over many generations (and hence, individuals and sexes). Second, many methods (including LDhat) assume that the population is at neutral equilibrium—an assumption that is frequently violated in nature which can lead to mis-inference (Dapper and Payseur 2018). Although methods exist that can take population demographic history into account when estimating recombination rates (*e.g.*, pyrho; Spence and Song 2019), our analyses are limited by the scarce data available for lizards in the genus *Sceloporus.* Namely, it is challenging to infer the demographic history of the two species in the first place without any prior knowledge of, for example, mutation rates, effective population sizes, or even which genomic regions to use for such inference as there are no genome annotations available that could be leveraged to select regions unaffected (or at least less affected) by selection [see Johri *et al.* (BioRxiv) for a discussion regarding statistical inference in population genomics]. This highlights the importance of developing further genomic resources for these important model organisms to improve our understanding of recombination rate evolution in squamates.

## Conclusion

As the field begins to gain a broader phylogenetic view of recombination rate variation and evolution, multiple hypotheses related to both the determinants and consequences of rate variation are anticipated to be better resolved. We here add two closely-related species of spiny lizards to this view. Despite similarities in karyotype, differences in recombination rate were observed at both the fine- and (to a lesser extent) broad-scale, highlighting the importance of including species-specific recombination maps in future population genomic analyses and genome-wide scans for targets of selection in the species. Moreover, our results suggest that major variation in the recombination landscapes of *Sceloporus* species with different chromosome counts remains to be discovered.

## Data availability

Supplementary Table S1 and Supplementary Figure S1 provide information on the population sampling. Supplementary Figures S2–S4 display the population SNP density, per-sample coverage, and number of variants per sample across scaffolds, respectively. Supplementary Figure S5 shows the relationships among individuals. Supplementary Figure S6 displays the genetic differentiation among individuals. Supplementary Figure S7 shows the cross-validation error in the ADMIXTURE models. Supplementary Figures S8 and S9 illustrate the variation in fine-scale recombination landscape within and between scaffolds in *S.jarrovii* and *S. megalepidurus*, respectively. The sequencing data from this study is available on NCBI's Sequence Read Archive under the BioProject designation PRJNA726723. The fine-scale recombination map is available at http://spfeiferlab.org/data.

Supplementary material is available at *G3* online.

## Supplementary Material

jkab402_Supplementary_Data
