# Supplementary material for: The recombination landscapes of spiny lizards (genus Sceloporus)
Source: G3 (Bethesda). 2021 Nov 22;12(2):jkab402. doi: 10.1093/g3journal/jkab402 (PMC9210290; doi:10.1093/g3journal/jkab402)
Supplement: jkab402_Supplementary_Data [file jkab402_supplementary_data.zip › GENETICS-G3-2021-403038-s01.docx]

| **species** | **ID** | **sex** | **# total reads** | **# mapped**  **reads** | **# non-primary**  **reads** | **# supple-mentary reads** | **coverage** |
| --- | --- | --- | --- | --- | --- | --- | --- |
| *S. jarrovii* | JAR6 | male | 123,818,878 | 119,491,971 | 0 | 9,528,756 | 9.8 |
|  | JAR13 | male | 134,048,924 | 129,260,049 | 0 | 10,306,558 | 10.6 |
|  | JAR15 | male | 131,011,874 | 126,306,299 | 0 | 10,060,651 | 10.4 |
|  | JAR16 | female | 126,600,732 | 122,111,634 | 0 | 9,774,759 | 10.0 |
|  | JAR17 | female | 115,707,808 | 111,451,992 | 0 | 8,900,151 | 9.2 |
|  | JAR19 | male | 126,256,350 | 121,670,837 | 0 | 9,747,579 | 10.0 |
|  | JAR50 | female | 142,399,250 | 137,523,333 | 0 | 11,039,105 | 11.4 |
|  | JAR53 | female | 147,290,818 | 142,213,098 | 0 | 11,482,724 | 11.7 |
| *S. megalepidurus* | MEG8 | male | 135,193,888 | 130,984,148 | 0 | 10,271,073 | 10.7 |
|  | MEG11 | male | 143,493,138 | 139,044,175 | 0 | 10,882,164 | 11.4 |
|  | MEG13 | male | 135,668,066 | 131,323,759 | 0 | 10,199,345 | 10.8 |
|  | MEG15 | female | 123,457,532 | 119,586,009 | 0 | 9,237,908 | 9.9 |
|  | MEG21 | female | 146,109,406 | 141,492,767 | 0 | 11,261,630 | 11.6 |
|  | MEG30 | male | 157,932,302 | 153,212,342 | 0 | 11,824,276 | 12.5 |
|  | MEG40 | female | 129,871,304 | 125,771,286 | 0 | 9,768,031 | 10.3 |
|  | MEG42 | female | 155,501,348 | 150,658,909 | 0 | 11,628,144 | 12.3 |

**Table S1. Samples, sequence alignment, and coverage statistics (assuming a genome size of 1.91Gb).**
