# Supplementary material for: The recombination landscapes of spiny lizards (genus Sceloporus)
Source: G3 (Bethesda). 2021 Nov 22;12(2):jkab402. doi: 10.1093/g3journal/jkab402 (PMC9210290; doi:10.1093/g3journal/jkab402)
Supplement: jkab402_Supplementary_Data [file jkab402_supplementary_data.zip › GENETICS-G3-2021-403038-s02.docx]

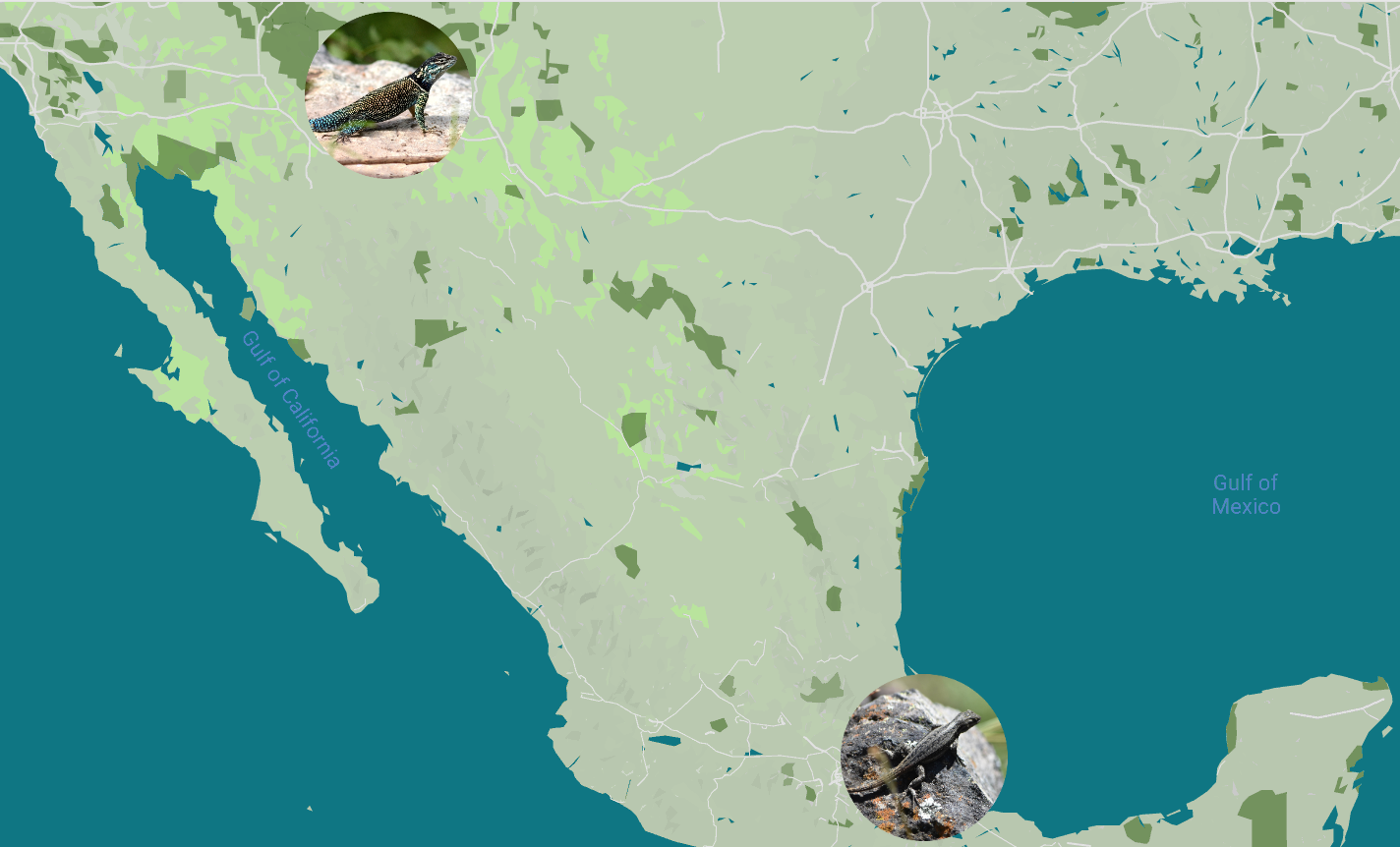


**Figure S1: Population Sampling.** *S. jarrovii* and *S. megalepidurus* individuals (n = 8 individuals per species; four males and four females) were captured in the field in Arizona, United States and in Veracruz, Mexico, respectively. Picture credits: squamatologist (*S. jarrovii*; distributed under a CC BY-NC-ND 2.0 license) and camamed (*S. megalepidurus*; distributed under a CC BY-NC 4.0 license).

*S. jarrovii S. megalepidurus*

**Figure S2: SNP density across scaffolds.** A large variation in SNP density is observed on the smallest scaffolds (blue), likely due to misaligned reads and artifactual variant calls. In order to limit the number of false positives in this study, analyses were restricted to scaffolds longer than 2Mb (*i.e.*, 88 scaffolds, highlighted in red).

*S. jarrovii S. megalepidurus*

**Figure S3: Per-sample coverage across scaffolds.** Scaffolds are displayed by decreasing length.

*S. jarrovii S. megalepidurus*

**Figure S4: Number of variants per sample across scaffolds.** Scaffolds are displayed by decreasing length.

*S. jarrovii S. megalepidurus*

**Figure S5: Relationships among individuals.** The software KING (Manichaikul *et al.* 2010) was utilized to estimate pairwise kinship coefficients. Coefficients larger than 0.354, 0.177–0.354, 0.0884–0.177, and 0.0442–0.0884 correspond to monozygotic twins, 1st-degree, 2nd-degree, and 3rd-degree relatives, respectively. Negative values indicate potential population structure.

**Figure S6: Principal component analysis.**

*S. jarrovii S. megalepidurus*

**Figure S7: Cross-validation error in the ADMIXTURE models.** Cross-validation error is plotted as a function of the ancestral source populations (*K*).

**Figure S8: Variation in the fine-scale recombination landscape within and between scaffolds in *S. jarrovii.***

**Figure S9: Variation in the fine-scale recombination landscape within and between scaffolds in *S. megalepidurus.***
